# BACs-on-Beads Technology: A Reliable Test for Rapid Detection of Aneuploidies and Microdeletions in Prenatal Diagnosis

**DOI:** 10.1155/2014/590298

**Published:** 2014-03-27

**Authors:** Sandra García-Herrero, Inmaculada Campos-Galindo, José Antonio Martínez-Conejero, Vicente Serra, Inés Olmo, Coral Lara, Carlos Simón, Carmen Rubio

**Affiliations:** ^1^IVIOMICS, Calle Catedrático Agustín Escardino 9, Paterna, 46980 Valencia, Spain; ^2^Obstetrics Unit, University Institute IVI Valencia, Plaça de la Policia Local 3, 46015 Valencia, Spain; ^3^Fundación Instituto Valenciano de Infertilidad (FIVI) e Instituto Universitario IVI/INCLIVA, Valencia, Spain; ^4^Department of Obstetrics and Gynecology, School of Medicine, Stanford University, CA, USA

## Abstract

The risk of fetal aneuploidies is usually estimated based on high resolution ultrasound combined with biochemical determination of criterion in maternal blood, with invasive procedures offered to the population at risk. The purpose of this study was to investigate the effectiveness of a new rapid aneuploidy screening test on amniotic fluid (AF) or chorionic villus (CV) samples based on BACs-on-Beads (BoBs) technology and to compare the results with classical karyotyping by Giemsa banding (G-banding) of cultured cells in metaphase as the gold standard technique. The prenatal-BoBs kit was used to study aneuploidies involving chromosomes 13, 18, 21, X, and Y as well as nine microdeletion syndromes in 321 AF and 43 CV samples. G-banding of metaphase cultured cells was performed concomitantly for all prenatal samples. A microarray-based comparative genomic hybridization (aCGH) was also carried out in a subset of samples. Prenatal-BoBs results were widely confirmed by classical karyotyping. Only six karyotype findings were not identified by Prenatal-BoBs, all of them due to the known limitations of the technique. In summary, the BACs-on-Beads technology was an accurate, robust, and efficient method for the rapid diagnosis of common aneuploidies and microdeletion syndromes in prenatal samples.

## 1. Introduction

Birth defects are responsible for many cases of infant mortality and morbidity around the world [1], with about seven percent of all neonatal deaths being caused by congenital anomalies [2]. It is estimated that six percent of these congenital defects are due to aneuploidies and nearly one in 200 newborns is affected [3].

These congenital defects are caused by chromosomal aneuploidies or monogenic disorders; nevertheless, environmental causes such as fetal infections, environmental teratogens, or micronutrient deficiencies could be hidden by the low percentages for these factors. In fact, most of these defects are due to the combined effects of environmental and genetic factors [2].

The risk of fetal aneuploidies is usually estimated based on high resolution ultrasound scans combined with biochemical determinations in maternal blood samples. The most important biochemical markers measured in the first trimester of pregnancy are the free beta-subunit of human chorionic gonadotropin (f*β*-hCG) and pregnancy-associated plasma protein A (PAPP-A), and the most important ultrasound marker is the measurement of nuchal translucency. In the second trimester, alpha-fetoprotein (AFP) and beta-human chorionic gonadotropin (*β*hCG) are also measured [2, 4]. In fact, second-trimester screening has been discontinued lately because it resulted in the worst detection rates (around 70% estimated detection rates for a 5% false positive rate for Down syndrome). Nevertheless, second-trimester screening would be only used when women are attending to the first medical consultation after 13 weeks of pregnancy. The current trend focuses on the first-trimester screening (11–13 weeks), with a hormonal measurement in 9-10 weeks (bhCG + PAPP-A) and nuchal translucency and other echography parameters measurements in 11–13 weeks, plus maternal age correction (around 85–90% estimated detection rates for a 5% false positive rate for Down syndrome). Furthermore, if we add other echographic findings as nasal bone, ductus venosus, and tricuspid blood flow, the estimated detection rates reach 93–96% for a 2.5% of false positive rate for Down syndrome. Given the established effectiveness of the first-trimester screening alone, its combination with the second-trimester screening would not increase detection rates [5–7]. If this risk is higher than 1/250, invasive procedures (CV sampling or amniocentesis) are recommended [8].

Fetal chromosomal analysis has traditionally been performed using Giemsa banding (G-banding) on cultured cells in metaphase, and it is considered the gold standard detection method [9, 10]. Although the accuracy and reliability of this technique are very high, 99.4–99.8% and 97.5–99.6% for amniocentesis and CV, respectively [11, 12], the main disadvantage is that the prenatal tissue must be cultured for several days prior to analysis, whereas in conditions like abnormal ultrasound findings or where there is maternal anxiety a much quicker diagnosis would be useful. For these reasons, a rapid test to discard the presence of the most common aneuploidies (13, 18, 21, X, and Y) in live born infants is very desirable [13].

The most common rapid molecular methods for prenatal aneuploidy detection are fluorescence* in situ *hybridization (FISH) and quantitative fluorescent polymerase chain reaction (QF-PCR) [14]. These two methods allow the detection of whole chromosome aneuploidy for chromosomes 13, 18, 21, X, and Y. Over the last three years we have introduced a new rapid prenatal diagnostic test called Prenatal-BoBs into our laboratory. This test is based on BACs-on-Beads technology, using BAC (bacterial artificial chromosome) clones attached to dyed microspheres. The advantage of this test compared to previous ones is that this multiplex assay not only includes markers for detecting aneuploidy in chromosomes 13, 18, 21, X, and Y but also contains markers for detecting nine microdeletion syndromes (DiGeorge, Williams-Beuren, Prader-Willi, Angelman, Smith-Magenis, Wolf-Hirschhorn, Cri du Chat, Langer-Giedion, and Miller-Dieker syndromes, Table 1 [15]). The microdeletion syndromes analyzed by Prenatal-BoBs were selected following these inclusion criteria: syndromes with a high relative prevalence (1/4000 to 1/200,000), significant morbidity/mortality, mild or unspecific ultrasound findings, strongly genotypic and phenotypic correlation, and deletions typically too small to be detected on a standard karyotype [15–17].

In recent years, microarray-based comparative genomic hybridization (aCGH) has been refined to determine chromosomal changes at progressively higher resolutions. In contrast to rapid aneuploidy detection, aCGH represents a comprehensive, genome-wide strategy to obtain chromosome copy-number information and, compared with conventional karyotyping, it is rapid, less labor-intensive, and readily amenable to automation. Being a genome-wide screening technique, its major advantage is that it has a vastly improved resolution compared to traditional karyotyping. The use of aCGH is recommended in concert with genetic counseling as an adjunct tool in prenatal cases where fetuses present abnormal ultrasound findings and a normal conventional karyotype, as well as in cases of fetal decease with congenital anomalies where a conventional karyotype cannot be obtained. However, its advantage is also its main handicap: high resolution allows the detection of a higher percentage of abnormalities than in a conventional karyotype but may also pick up unexpected findings and/or variants that are of unknown clinical significance [18].

In this paper we describe our experience with this new technology in prenatal diagnosis from AF and CV samples, comparing Prenatal-BoBs results with those obtained by conventional karyotypes. Furthermore, in a subset of samples, we compare Prenatal-BoBs results with the results from aCGH in cases in which the test was prescribed. The array platform employed in the present study was the CytoChip Focus (BlueGnome, UK) based on BACs technology. This array detects aneuploidies and also analyses 143 chromosomal regions of known clinical significance.

## 2. Materials and Methods

### 2.1. Human Samples for Prenatal-BoBs Analysis

Between May 2010 and June 2013, our laboratory performed 364 Prenatal-BoBs tests, 321 samples were from AF and 43 from CV samples.

Sample collection and transport to our laboratory were performed at room temperature. A minimum of 5 mL of AF was required and DNA extraction was performed immediately after reception, or alternatively the sample was gently centrifuged, the supernatant was discarded, and then pellet was stored at −20°C until extraction. CV samples were also processed immediately or stored at 4°C until DNA extraction. DNA was extracted manually from 5-4 mL of AF, or for CV samples, from a microscopically selected, entire, native villous tree or 3–5 mg of tissue, according to the manufacturer's recommendations (QIAamp DNA Mini Kit, Qiagen, Inc., Chatsworth, CA, USA). A portion of the AF or CV sample was always reserved for conventional culture and karyotyping. Furthermore, 14 samples were additionally analyzed by aCGH (BlueGnome, UK).

For conventional karyotyping, AF or CV samples were cultured for 10–12 days with 5% CO_2_ at 37°C under sterile conditions. A minimum of 20 metaphase cells were analyzed with minimum resolution level of 550 bands.

### 2.2. BACs-on-Beads Technology

Prenatal-BoBs is a multiplex, bead-based suspension array using microspheres that are internally dyed with a combination of two spectrally distinct infrared and red fluorochromes which can produce more than 100 specific spectrums. Each bead is coupled to DNA amplified from BACs (a total of 83 different PCR-amplified BAC clones) and analyzed using a Luminex cytometric acquisition system with two separate lasers (Luminex Corp., Austin, Texas) equipped with xPonent 3.1 software (Perkin Elmer, Turku, Finland). Experiments with acceptable quality control parameters had more than 50 beads/BACs analyzed alongside both male and female samples which were included as reference DNAs [19]. Prenatal-BoBs assesses 75 chromosomal regions involving chromosomes 13, 18, 21, X, and Y as well as the nine previously mentioned microdeletion syndromes.

**Figure 1:**
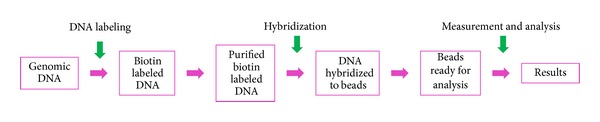
BACs-on-Beads assay flowchart.

Briefly, genomic DNA was extracted, labeled, purified, hybridized to BACs-on-Beads probes, bound to the reporter molecules (streptavidin-phycoerythrin), and washed. Thereafter the fluorescence signals were measured and the results analyzed (Figure 1). Once the DNA was extracted, it was amplified with a primer solution, labeled by enzymatic incorporation of biotinylated nucleotides, and purified using a PCR purification kit. Then, it was incubated overnight with BAC clones attached to dyed beads, after which the hybridized beads were transferred onto a filter plate and washed again. After washing, the beads were incubated with a reporter that binds to biotinylated DNA and then washed and resuspended for measurement according to the protocol recommended by the manufacturer.

**Figure 2:**
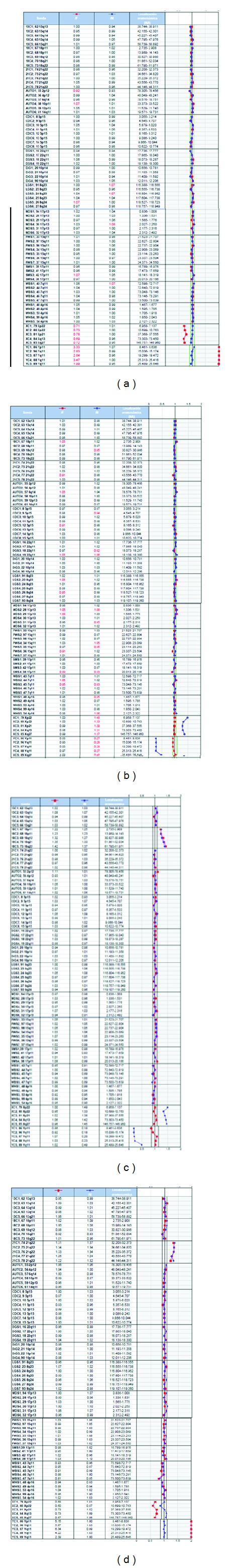
Examples of Prenatal-BoBS results: (a) male normal fetus, (b) female normal fetus, (c) female fetus with a trisomy of chromosome 18, and (d) male fetus with a trisomy of chromosome 21.

The relative amount of DNA bound to the beads was determined using a Luminex 100/200 instrument system with xPONENT 3.1 and BoBsoft V2 analysis software that produces graphical ratio line-plots and a bar graph for each sample. A sample was defined as “duplicated/deleted” in a chromosome locus when the fluorescence in the test was higher/lower than that in the reference. Single copy gains and losses generate ratios ranging from 1.3 to 1.4 and from 0.6 to 0.8, respectively (Figure 2) [16].

### 2.3. Human Samples for Microarray-Based Comparative Genomic Hybridization Analysis

Approximately 7–10 mL of AF was cultured for 10–12 days with 5% CO_2_ at 37°C under sterile conditions. Once a cell monolayer was obtained, it was trypsinized and DNA was extracted. DNA extraction from CV samples did not require previous cell culture. DNA was extracted using (QIAamp DNA Mini Kit, Qiagen, Inc., Chatsworth, CA, USA) according to the manufacturer's protocol and the concentration and purity of the extracted DNA were measured with a NanoDrop spectrophotometer (NanoDrop Technologies, Inc.).

Following DNA extraction, the test and reference DNA were cohybridized to the array. Briefly, 400 ng of patient and reference DNA was labelled by random priming with Cyanine 3 and Cyanine 5 fluorescent dyes, respectively. DNA was then hybridized on the arrays (CytoChip Focus Constitutional, BlueGnome Ltd., UK) over night at 48°C. Once the arrays were washed, images were acquired using a PowerScanner microarray scanner (Tecan, Switzerland) and the image files were analyzed with the BlueFuse microarray software package (BlueGnome, UK).

## 3. Results

### 3.1. BACs-on-Beads Results

Finally, 364 Prenatal-BoBs tests were performed (321 in AF and 43 in CV samples), obtaining conclusive results in 362 cases (99.45%). Conventional G-band karyotyping was also performed and the results from cultured cells were obtained in 335 cases out of the initial 364 samples (92.03%). Karyotype failures were mainly attributed to cell culture failures, with an increased percentage of growth failure in CV samples (41.86% in CV versus 3.43% in AF samples; Fisher's exact test, *P* < 0.001).

Using Prenatal-BoBs tests, we found normal results in 309 AF (96.26%) and 35 CV (81.40%) samples. The most frequent chromosome abnormality found was trisomy 21 (*n* = 13, representing 81.25% of the total abnormal findings), but we also found an abnormal result corresponding to Cat Eye microduplication syndrome (22q11). The rate of concordance with the conventional cytogenetic method was 98.51% (*n* = 335). Three of the five samples (1.49%) that did not show concordance corresponded to chromosome abnormalities which were not detectable by Prenatal-BoBs: two Robertsonian translocations [45,XY,der(13;14)(q10;q10) and 46,XX/45,XX,der(13;14)(q10;q10)] and one reciprocal translocation [46,XY(3;4)(p22;q21)]. The other two samples correspond to polymorphisms (46,XY,15p+++ and 46,XYqh). Summarized results are shown in Table 2.

### 3.2. Microarray-Based Comparative Genomic Hybridization Results

**Figure 3:**
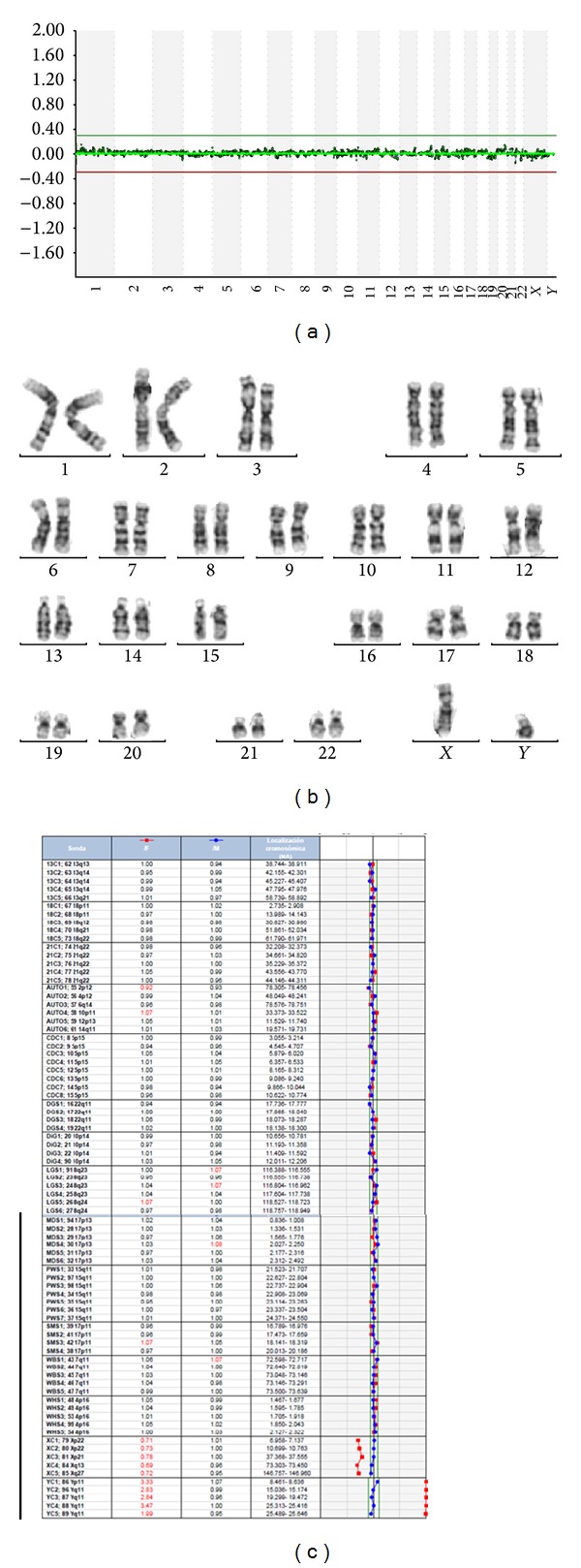
Male normal fetus: (a) CGH array focus (b), conventional karyotype, and (c) Prenatal-BoBS results.

Fourteen patients also underwent aCGH testing in addition to Prenatal-BoBs and conventional G-banding karyotyping: 12 AF and 2 CV samples. In all of them, results were concordant with the karyotype and the Prenatal-BoBs test (Figure 3), including Cat Eye syndrome detected by BoBs and confirmed by CGH array platform.

## 4. Discussion

In this work we compared the results of a rapid aneuploidy test with those obtained with conventional karyotypes. Our results showed that Prenatal-BoBs is a reliable, robust, and efficient method for the rapid diagnosis of common aneuploidies in prenatal samples.

Despite the fact that other rapid aneuploidy tests, such as QF-PCR or FISH, have been used for several years [20], there is no consensus as to whether women at increased risk for trisomies 13, 18, or 21 should be offered stand-alone rapid aneuploidy tests or conventional G-banding karyotyping [20].

The major advantages of rapid aneuploidy tests include fast reporting (within 24 to 48 hours) and earlier anxiety relief. This fact is particularly important in the process of medical decision-making in cases of maternal anxiety, where there are fetal abnormalities found in the ultrasound examination, or if there are very few days to make a decision regarding a termination of pregnancy [21, 22]. However, about 15–30% of potential chromosome abnormalities that are detected by karyotyping would be missed using these tests in prenatal diagnosis, although this percentage is lower bearing in mind that many of those abnormalities die* in utero* [23]. It has been estimated that for approximately every 1000 amniocenteses performed, up to four potentially clinically significant chromosomal abnormalities may be missed with rapid aneuploidy tests (e.g., balanced translocations, the presence of marker chromosomes, or low grade mosaicism) [21, 22]. Therefore, mainly for this reason (loss of chromosome abnormality information in chromosomes other than 13, 18, 21, and X, and/or balanced rearrangements), clinicians and patients must balance the benefits and drawbacks of conventional karyotyping versus rapid prenatal tests such as Prenatal-BoBs.

Currently, there are two main trends of opinion in this issue: either rapid aneuploidy tests should replace karyotyping for indications such as positive Down's syndrome screening, in cases of advanced maternal age, and in pregnancies without ultrasound abnormalities [21–23] or both rapid aneuploidy testing and karyotyping should be carried out [20]. Those in favor of the first option generally argue that the error rates using a rapid aneuploidy test are acceptable [24, 25] and that a final clinical decision based on full karyotyping should mostly be confined to a selected group of women with specific indications [9].

Although rapid aneuploidy tests indicate that only 1% of all invasive prenatal samples have an undetected chromosomal abnormality, a third of these can have a significant risk of serious phenotypic consequences [20], and so some authors recommend conventional karyotyping to try to avoid the potential devastating medical, emotional, and financial consequences that an infant born with severe handicaps could have for parents and live births.

In contrast to other rapid aneuploidy tests, Prenatal-BoBs presents some special features: it is a CE-IVD certified kit with the ability to detect nine microdeletion syndromes with a high specificity (>99%; false positive rate <1%) and sensitivity (>98%; false negative rate <2%) [15, 19]. More specifically, when compared to FISH, BACs-on-Beads technology results are more objective, easier to interpret, and its protocol is robust, fast to implement in the laboratory, and amenable to automation [15, 19]. Moreover, the concordance of aneuploidies detected between karyotyping and Prenatal-BoBs was nearly 100%. An estimation of the rate of concordance for microdeletion syndromes was established in the few samples in which aCGH was prescribed and showed 100% concordance for the microdeletion syndromes included in the Prenatal-BoBs.

In the near future, the advent of massively parallel sequencing is likely to augur a big change in clinical practice in noninvasive prenatal testing (NIPT) [26]. NIPT for aneuploidy using cell-free DNA in maternal blood plasma is revolutionizing prenatal screening and diagnosis scenarios. Clinical trials have demonstrated the efficacy of NIPT for the same chromosomal abnormalities that the rapid aneuploidy testing panel analyzes [27–29]. The main advantage of this new approach is the reduction in the need for invasive prenatal diagnostic practices (such as amniocentesis) to obtain biological material from fetus, thus minimizing the risk of iatrogenic miscarriages [30, 31]. Nevertheless, this is not yet a diagnostic tool but rather a screening test, and so positive NIPT results must still be confirmed using invasive techniques.

## 5. Conclusions

BACs-on-Beads technology was an accurate, robust, and efficient method for the rapid diagnosis of common aneuploidies and microdeletion syndromes in prenatal samples. High concordance of detected aneuploidies was observed between karyotyping and Prenatal-BoBs test.

## Figures and Tables

**Table 1:** Description of the aneuploidies and microdeletion syndromes included in the Prenatal BoBS kit.

Syndrome	Frequency of occurrence	Lifespan	Mental retardation	Severe medical symptoms
Down syndrome (21)	1/750–800	50 years	Mild to moderate	−/+
Patau syndrome (13)	1/6,000	4 days	Severe	++
Edwards syndrome (18)	1/10,000	2.5 days	Severe	++
Triple X syndrome (XXX)	1/1,000	Normal	No	−
Klinefelter syndrome (XXY)	1/500–1,000	Normal	No	−
XYY syndrome (XYY)	1/1,000	Normal	No	−
Turner syndrome (X0)	1/2,500	Slightly reduced	Mild to moderate	−/+
Wolf-Hirschhorn (4p16, 3)	1/50,000	Limited	Moderate to severe	+
Cry du Chat (5p15, 3-p15, 2)	1/15,000–50,000	Normal	Moderate to severe	−/+
Williams-Beuren (7q11, 2)	1/7,500–20,000	Reduced	Mild to moderate	−/+
Langer-Giedion (8q23-q24)	unknown	Normal	Mild to severe	−/+
Prader-Willi (15q11-q12)	1/10,000–30,000	Normal	Mild	−/+
Angelman (15q11-q12)	1/12,000–25,000	Normal	Severe	−/+
Miller-Dieker (17p13, 3)	1/100,000–300,000	Reduced	Profound	−/+
Smith-Magenis (17p11, 2)	1/25,000–50,000	No data	Mild to moderate	−/+
DiGeorge (10p14)	1/4,000–5,000	Reduced	Mild to moderate	+
DiGeorge (22q11, 2)	1/2,000–4,000	Reduced	Mild to moderate	+

The severity and type of the symptoms are represented from − (in cases where symptoms range from none to mild) to ++ (for those ranging from moderate to severe). The information in this table was adapted from the following resources: http://www.orpha.net, http://www.nlm.nih.gov, and http://www.rarechromo.org.

**Table 2:** Results obtained with Prenatal-BoBs—karyotype-analysis in both AF and CV samples.

	CV					AF				
	Abnormal	Normal		NI	Total	Abnormal	Normal		NI	Total
BoBs	7	35		1	43	11	309		1	321

Karyotype	5	20		0	25	11	299		1	310

Karyotype findings	0	2	46, XX/45, XX, der (13; 14) (q10; q10)	0		0		46, XY, 15p+++	0	
			46, XY (3; 4) (p22; q21)				3	45, XY, der (13; 14) (q10; q10)		
								46, XYqh+		

CV: chorionic villus samples; AF: amniotic fluid; NI: noninformative.
